# The role of *Staphylococcus aureus* enterotoxin B in chronic rhinosinusitis with nasal polyposis

**DOI:** 10.1186/s12964-022-00839-x

**Published:** 2022-03-09

**Authors:** Zahra Chegini, Mojtaba Didehdar, Amin Khoshbayan, Jafar Karami, Milad Yousefimashouf, Aref shariati

**Affiliations:** 1 Department of Microbiology, School of Medicine, Hamadan University of Medical Sciences, Hamadan, Iran; 2 Department of Medical Parasitology and Mycology, Arak University of Medical Sciences, Arak, Iran; 3 Department of Microbiology, School of Medicine, Iran University of Medical Sciences, Tehran, Iran; 4 Molecular and Medicine Research Center, Khomein University of Medical Sciences, Khomein, Iran; 5 Department of Medical Laboratory Sciences, Khomein University of Medical Sciences, Khomein, Iran; 6 Department of Medical Laboratory Sciences, Faculty of Paramedical, Borujerd Branch, Islamic Azad University, Borujerd, Iran

**Keywords:** Chronic rhinosinusitis, Type 2/Th2 pathway, *Staphylococcus aureus*, Enterotoxin B, Nasal polyps

## Abstract

CRS with nasal polyps (CRSwNP) is a multifactorial disease, and various etiological factors like bacterial superantigens are known to develop this disease. Recent studies reported that *Staphylococcus aureus* nasal colonization was detected in 67% of the patients with CRSwNP. Moreover, it was reported that specific IgE against *S. aureus* enterotoxins are discovered in almost half of the nasal tissue homogenates from nasal polyps. Thus, investigations have highlighted the role of staphylococcal enterotoxins, especially enterotoxin B (SEB), in pathogenesis of CRSwNP. The destruction of mucosal integrity was reported as a main SEB-related pathogenic mechanisms in CRSwNP. SEB activates Toll Like Receptor 2 and triggers the production of pro-inflammatory cytokines; furthermore, it induces reactive oxygen species and endoplasmic reticulum stress-induced inflammation that may cause epithelial cell integrity disruption and enhance their permeability. SEB-induced Type 2/Th2 pathway results in degranulation of eosinophils, cationic proteins production, and localized eosinophilic inflammation. Furthermore, SEB may be involved in the expression of RORC and HIF-1α in Tregs and by maintaining the inflammation in sinonasal mucosa that could have a main role in the pathogenesis of nasal polyposis. Different in vitro findings were confirmed in animal studies; however, in vivo analysis of SEB-induced nasal polyps and CRS remains unfulfilled due to the lack of appropriate animal models. Finally, after elucidating different aspects of SEB pathogenesis in CRSwNP, therapeutic agents have been tested in recent studies with some encouraging results. The purpose of this article is to summarize the most important findings regarding SEB-induced CRS and nasal polyposis.

**Video Abstract**

**Supplementary Information:**

The online version contains supplementary material available at 10.1186/s12964-022-00839-x.

## Background

Chronic rhinosinusitis (CRS), an inflammatory disease affecting the nasal cavity and paranasal sinuses, is one of the most common chronic diseases afflicting humans. Symptoms include facial pressure and pain, nasal obstruction and discharge, and prolonged anosmia (over 12 weeks). This disease has a remarkable socioeconomic effect that affects up to 14 and 10.9% of Americans and the European population, respectively [1, 2]. CRS is classified into two subtypes based on several clinicopathological characteristics: CRS without nasal polyps (CRSsNP) and CRS with nasal polyps (CRSwNP) [3]. T helper 2 (Th2)-based cytokine profile and eosinophilic infiltration were reported in about 80% of CRSwNP. Nonetheless, types 1 and 3 of inflammation characterized by increased interleukin (IL)-6, IL-8, IL-17 and TNF-a levels, and neutrophil infiltration were more strongly associated with the CRSsNP [3–6].

Nasal polyps, benign inflammatory masses, arise from any portion of the mucosa of the paranasal sinuses and nose that lead to chronic nasal obstruction. These polyps are considered to be a CRS subgroup, and clinical diagnosis is made on the basis of the polyps visualization in the nasal cavity and the presence of sinonasal symptoms for more than three months [7]. Although the precise mechanism for the development of polyps remains unclear, recent investigations propose a complicated dysregulation of the interface of innate and adaptive immunity. The nasal mucosa indicates histologic remodeling that is determined by epithelial-mesenchymal transition with fibrin deposition and goblet cell hyperplasia [8, 9]. The results of other studies have also shown association from sinonasal epithelial barrier weaknesses. Injured epithelium from proteases, irritants, and infection leads to enhanced Th2-promoting cytokines production. This type 2 inflammatory pattern is identified by a high presence of T-helper 2 (Th2) cells, mast cells, and eosinophils [10]. Different cytokines such as IL-4, IL-5, and IL-13 have also been reported to drive the immunologic pathways central to nasal and sinus polyp’s pathophysiology. Unlike CRSsNP patients, high levels of IL-5 were detected in patients with polyps [11]. Collectively, CRSwNP patients have complex pathophysiologic mechanisms that illustrate heterogeneity between patients [9, 12].

Various immunopathological processes with persistent inflammation in the mucosal surface lead to CRS. As a result, CRS is a multifactorial inflammatory disorder whose precise pathogenesis remains unknown. However, different etiological factors such as *Staphylococcus aureus* enterotoxins (mainly Enterotoxin B (SEB)) have been reported for this disease. *S. aureus* is one of the common human bacteria often detected in the normal nasal microbiota of healthy individuals. A previous study reported that *S. aureus* nasal colonization was detected in 67% of patients with CRSwNP [13, 14]. Moreover, it was reported that specific IgE against *S. aureus* enterotoxins are discovered in almost half of the nasal tissue homogenates from nasal polyps [15].

Under certain conditions, this bacterium can colonize the nasal mucosa, and this phenomenon can facilitate its invasion into the subepithelial regions [16, 17]. SEB damages and remodels tissue by inhibiting regulatory T cells, increasing Th2 cytokine production, and enhancing eosinophil and mast cell functions [18, 19]. Furthermore, SEB can lead to the production of endoplasmic reticulum stress and reactive oxygen species (ROS) in epithelial cells of patients with CRSwNP [20]. In this regard, the results of recent investigations showed a correlation between *S. aureus* enterotoxins and different disorders such as allergic rhinitis, atopic dermatitis and asthma [21, 22].

Although researchers have reported disparate and inconsistent mechanisms of CRS pathogenesis, our understanding of this disease is still evolving, and the role of Staphylococcal superantigens, particularly SEB, in the pathogenesis of CRS and nasal polyposis is not fully understood. Thus, in the present review paper, we have attempted to clarify the possible underlying pathogenic mechanisms of SEB in triggering CRS and nasal polyposis.

## Colonization of upper airways by *S. aureus*

Approximately 260 million people reported viral or bacterial rhinosinusitis each year, with acute bacterial sinusitis accounting for approximately 0.5 to 2% of cases [23, 24]. *S. aureus* is among one of the most common bacteria related to CRS. To this end, one study from the USA reported that 10 (50%) and 14 (66.7%) of the swab samples from patients undergoing outpatient endoscopic sinus surgery were detected as *S. aureus* by culture and pyrosequencing, respectively. The authors also reported that CRS patients had significant differences in microbial communities compared to non-CRS patients with more abundant *S. aureus* [25]. A meta-analysis by Ou et al. investigated the possible association between *S. aureus* and CRSwNP among 12 studies from all continents consisting of 340 cases and 178 controls. The *S. aureus* culture-positive rate was remarkably higher in the CRSwNP group than in the control group. Additionally, *S. aureus* superantigens and specific IgE were remarkably higher than in the control group [26].

Furthermore, a recently published study from China found that all 36 patients with CRSwNP had a significant increase in SEB and SEA levels in the nasal tissues (turbinates and polyps). Thus, the authors suggest that *S. aureus,* especially in the Chinese population, could have the leading role in the pathogenesis of CRSwNP [27]. Moreover, another study from Belgium investigated the presence of *S. aureus* enterotoxin genes in isolated bacteria from the middle meatus of nasal polyp and control patients. In 75% of all isolates, at least one of the enterotoxins genes was detected, but there was no difference between strains collected from patients and the control group [28].

Moreover, several studies focus on the presence of *Staphylococcus* enterotoxin-IgE in a comparison between CRS patients and the control group. In this respect, some studies indicated that in the Caucasian population, enterotoxins could act as superantigens in the eosinophilic subgroup of CRSwNP; also, 37–50% of CRS patients had *S. aureus* enterotoxin specific IgE positive [29, 30]. A study from Japan compared the antibody level between CRSwNP, CRSsNP, and control group, and results demonstrated a remarkably higher level of SAE-IgE in the CRSwNP group. Also, this study suggests a similar cytokine profile in CRSwNP patients in Japan and European CRS [31]. Furthermore, a study from China reported that in the eosinophilic and non-eosinophilic CRSwNP groups, the increase of total IgE, SEA, and SEB was observed compared to the control group [32].

Additionally, a multicenter study demonstrated that the level of *S. aureus* enterotoxin-specific IgE is significantly higher in CRSwNP patients from Europe, Australia, and Japan. In contrast, in Southeast Asia, this number was remarkably lower, suggesting the variable effect of *S. aureus* in the pathogenesis of nasal polyp [33, 34]. To this end, a recent study from South Korea investigated the specific IgE-SEB among 965 patients. The study results demonstrated that IgE-SEB level was higher in CRS patients than non-CRS patients. Interestingly, there were no differences in SEB-IgE level or rate among CRSwNP and CRSsNP groups [35].

In contrast to the studies mentioned above, a study from Germany analyzed the detection rate of *S. aureus* between the three groups of CRSwNP, CRSsNP, and control. The results demonstrated a higher prevalence of *S.aureus* in CRS patients with or without nasal polyps than in controls [36]. Furthermore, another study from China reported a low rate of the *S. aureus* enterotoxin superantigens with patients with CRSwNP [37]. Similar results were reported from two separate studies from Lebanon and Switzerland [38, 39]. Chakhtoura et al. demonstrated that among 34 collected samples, only three (8.8%) were *S. aureus* positive, and one was detected as a carrier of enterotoxin B [38]. Moreover, Heymans et al. did not report any correlation between staphylococcal exotoxin genes and the presence or severity of CRS with or without nasal polyposis [39].

As a result, SEB was detected in patients with a different subtype of sinusitis; however, there was a discrepancy in the results obtained from cases worldwide. Although SEB is a known risk factor for CRS, the findings make it impossible to describe a specific SEB prevalence in patients with different subtypes of CRS, and there is a clear need for additional research.

## The destruction of mucosal integrity by SEB

By clearing foreign molecules from the sinonasal mucosa via mucociliary clearance and secreting antimicrobial peptides, the mucosa's innate barrier defense creates a physical impediment and protects the host from an external microorganism. Apical junctional complexes, like tight junctions (TJs), connect airway epithelial cells to each other and decrease para-cellular transport of various ions and macromolecules. In this regard, pathogens reach the subepithelial regions by cleaving TJ proteins and breaching epithelial barriers [40, 41]. Therefore, an undamaged epithelial barrier is essential for preventing and inhibiting airway diseases. Numerous studies have reported varying effects of SEB on epithelial barrier function, and we will discuss this interaction in this section.

Martens et al. applied SEB to the nasal polyp epithelial cells of CRSwNP patients, and their results demonstrated that SEB boosts FD4 permeability and declines trans-epithelial electrical resistance (TEER) in a dose-dependent manner. Overall, SEB disrupts epithelial cell integrity and inhibits the expression of zonula occludens (ZO)-1 and occludin proteins. Moreover, SEB activated TLR2 and led to the production of IL-6 and IL8. Stimulation of air–liquid interface culture with these cytokines decreased epithelial integrity. Notably, inhibition of TLR2 signaling prevented SEB-induced barrier disruption. The results of the animal model study also confirm these findings. In this regard, the authors stimulated control mice nose with 50 µl of SEB (Endonasal) and observed that SEB compromised barrier function and diminished expression of ZO-1 and occludin mRNA. On the other hand, the mentioned factors remained unaltered in *tlr2* -/- mice [40]. Another investigation also reported that factors secreted by *S. aureus*, either a combination of secreted factors or a protein-macromolecule, result in disruption of TJ in Human nasal epithelial cells grown at air–liquid interface cultures [42].

Perturbations of endoplasmic reticulum (ER) homeostasis, including higher protein synthesis, disrupted ER redox status, and misfolded proteins accumulation, could lead to the ER stress response. Furthermore, inflammation generates Reactive Oxygen Species (ROS), induces ER stress, and is involved in the development of nasal polyposis [43]. In this regard, Kim et al. reported a possible correlation between SEB and endoplasmic reticulum ER stress in the pathogenesis of CRSwNP [44]. Their findings showed that membrane-derived vesicles might deliver SEB to the cytosol of epithelial cells, and SEB may mediate ER stress response [44]. SEB is known as an essential inflammation inducer in human nasal epithelial cells. As mentioned, there is a link between inflammation and ER stress response that results in tissue repair or control tissue damage. However, the final result of ER stress-induced inflammation is detrimental or protective outcomes [45, 46]. Therefore, the authors proposed that SEB-induced ER stress may correlate with the progression of nasal polyposis. Additionally, SEB induces ROS production from eosinophilic and non-eosinophilic polyps compared to the healthy mucosa. This finding suggested that ROS produced by SEB may be involved in ER stress responses [44].

Notably, different allergens and environmental pollutants triggering the ER stress response and ROS production may conduct mitochondrial dysfunction in airway epithelial cells [47]. Furthermore, oxidative phosphorylation in the mitochondrial electron transport chain produces Mitochondrial ROS (mtROS) and Mitochondria using manganese-dependent superoxide dismutase (Mn-SOD) control mtROS. Nonetheless, cigarette smoke and external pathogens decrease Mn-SOD activity and cause ROS production [48, 49]. In this respect, Yoon et al. reported increased mtROS level in human nasal epithelial cell line after SEB exposure, related to the formation of nasal polyps [20]. In fact, mtROS could regulate the mitochondrial structure and function, generate a malfunctioned cycle, and develop disease pathophysiology [50]. As a result, SEB can generate a high level of mtROS and causes bioenergetics and morphological changes in mitochondria. These results suggest another aspect of SEB pathogenesis that may contribute to the development of CRSwNP; however, additional research is required [20].

Thus, it appears as though SEB can compromise the integrity of TJs and the epithelial barrier by activating TLR2 and producing pro-inflammatory cytokines. This phenomenon causes epithelial cell integrity disruption and enhances their permeability. In this regard, disruption of the TLR2 signaling pathway could be a novel therapeutic approach for inhibition of the pathophysiological consequences of SEB on inflammation in CRSwNP. Additionally, SEB induces ROS and ER stress-induced inflammation and may contribute to epithelial cell dysfunction and morphological changes. However, further animal and clinical studies are needed for the exact identification of these processes.

## The interaction between the SEB and immune system

Scientists have suggested underlying pathogenic mechanisms of SEB in CRS and nasal polyposis disease. This section reviews the enterotoxin's most critical interactions with the immune system, which may result in the development of CRS and nasal polyposis.

### T cells

Recent studies demonstrating the presence of distinct pro-inflammatory mediators such as CRSwNP tissue IL-5, IL-17, and IFN-γ, myeloperoxidase, eosinophilic cationic protein, and IgE in homogenates of CRSwNP tissue support the concept of distinct T cell subsets and consecutive variations in inflammatory patterns in patients with CRS [11, 29, 51]. When activated by different antigens, T-helper lymphocytes, the central cells of the adaptive immune response, are converted into significant effecter subtype cells, including Th1, Th2, or Th17. These cells release a particular pattern of cytokines and trigger different immune pathways. In this regard, INF-γ, TNF-α, and macrophage activating factors produced by Th1target intracellular pathogens. Furthermore, Th2, by secretion of IL-4, IL-5, IL-10, and IL-13, generates humoral immune and allergic inflammatory responses [52]. Finally, Th17 by IL-17 secretion facilitates mucosal barrier maintenance, simplifies host defense against pathogen infection, and potently triggers autoimmune disorders and tissue inflammation [53].

In an examination, nasal polyps and adjacent non-polypoidal sinonasal tissues were collected from patients with CRSwNP. Furthermore, as control samples, sinonasal mucosa was isolated from patients undergoing trans-sphenoidal pituitary surgery. Afterward, control and nasal polyp samples were stimulated by SEB using a nasal explant model. Intriguingly, similar immune profiles skewed towards the Th2/Th17 pathway were detected in the same patient's non-polypoidal sinonasal mucosa and nasal polyps. In this regard, the expression of IL-1β and IL-6 genes were decreased when nasal polyps were challenged with SEB. However, these pro-inflammatory responses were not identified in control samples [54]. As a result, the authors suggested that non-polypoidal sinonasal mucosa may develop over time into frank nasal polyps. To this end, this area needs targeting with topical steroids for reduction of immune responses and to inhibit transformation into nasal polyps and prevent recurrence following surgical removal. Therefore, the ability of SEB in Th2 and Th17 pathways induction highlights the role of bacterial toxins in the promotion and sustained chronic inflammation [54, 55].

Moreover, Rha et al. demonstrated that in nasal polyps, the staphylococcal enterotoxin-related expanded CD4^+^ T cells are Th2. These cells can respond to epithelial-derived cytokines, eventually escalating the Th2 inflammation. Additionally, CD4^+^ T cells play an important role in type 2 inflammation and could act as a regulatory target in CRSwNP management [15].

Th2-polarizing cytokines pathogenic mechanisms in CRS were reported in different investigations. P-glycoprotein (P-gp), permeability glycoprotein, is an efflux pump that is able to manage the expression and transport of cytokine in the sinonasal mucosa [56, 57]. In this respect, Bleier et al. evaluated the association between P-gp and the secretion of IL-5, IL-8, and thymic stromal lymphopoietin (TSLP) following exposure to SEB. The results showed that P-gp protein is overexpressed in nasal polyp tissue in CRSwNP compared to CRS. Furthermore, exposure to SEB leads to the higher secretion of TSLP and IL-5 but not of IL-8 in the CRSwNP explants compared to the CRS explants. Notably, the inhibition of P-gp by zosuquidar trihydrochloride reduces cytokines secretion to baseline levels and suggests a prosecretory role for the efflux pump. Therefore, the higher expression of P-gp in nasal polyps could be related to the hypersecretion of TSLP and IL-5 in CRSwNP patients [58]. These results point to a new mechanism for Th2 promoting and maintaining response within CRSwNP; however, further studies to confirm this are required. In the end, it should be noted that Nasal fibroblast produces TSLP, and its expression is increased in nasal polyps and allergic rhinitis [59]. The findings of a recently investigations indicated that allergens could increase the production of TSLP in nasal fibroblasts, especially Nasal Polyp Fibroblasts (NPF) [60].

Furthermore, another study isolated Dispersed Nasal Polyp Cells (DNPCs) from CRSwNP patients and cultured them with SEB and fungal extracts from *Candida*, *Aspergillus*, and *Alternaria*. A significantly higher expression of IL-5, IL-13, and RANTES was detected in DNPCs after exposure to SEB compared to the fungal extract. These distinctions may be explained by the fact that fungal extracts elicit antigen-specific responses, whereas responses to SEB are superantigen responses [61]. Finally, in another examination, Peripheral Blood Mononuclear Cells (PBMCs) were collected from patients with asthma and CRS, and these cells were then cultured with a mixture of the specific airborne antigens (antigen-related asthma) in the presence or absence of SEB. The authors observed high levels of IL-4 from isolated PBMCs when stimulated by specific antigens. Therefore, it is hypothesized that IL-4 is directly secreted by antigen-activated Th2 cells. While IL-4 production increases in cultured PBMCs during the first four days, it gradually declines after that, even in the presence of specific antigens. Nevertheless, when SEB was added, IL-4 production increased again in the PBMCs of CRS-asthma patients, but not in those with CRS or asthma alone or healthy controls. As a result, SEB may induce persistent Th2 responses in individuals with low airway hypersensitivity associated with sinusitis. In this regard, removing infectious foci, especially in the airway, could lead to the management of CRS and asthma [62].

Noteworthy, Th2-polarizing cytokines such as IL-4 and IL-5 trigger IgE production from plasma cells. Subsequently, eosinophils covered with IgE could bind to SEB and lead to release of cationic proteins, eosinophils degranulation, and localized eosinophilic inflammation [6]. To this end, a positive association between eosinophil cationic protein and antigen-specific IgEs in CRSwNP patients was reported [63]. In this manner, SEB-specific IgEs had a higher prevalence rate in the sinus mucosa, but not in serum; therefore, staphylococcal enterotoxins-specific IgEs prompted local inflammation [63–65]. Moreover, Zhang et al. also reported that mucosal IgE in nasal polyp tissue are functional and able to activate mast cells; specific IgE in nasal polyp tissue can be found independently of their presence in serum [66].

Finally, dispersed nasal polyp cells are reported to produce IL-5 and IL-13 when exposed to SEB [61]. These cytokines cause eosinophilic inflammation by managing eosinophil differentiation, recruitment, and survival [61]. Interestingly, the results of recent study showed that staphylococcal enterotoxins have an ability to induce basophil activation in severe asthma; however, further studies will be necessary to validate these findings [67]. Hence, IgE-mediated hypersensitivity to SEB could act as a considerable pathogenic mechanism that drives localized eosinophilic inflammation in CRSwNP patients, which may be relevant to the pathogenesis of CRS and nasal polyposis.

It is worth mentioning that the interaction of SEB with other cytokines could have a possible role in CRSwNP, as shown in Table 1.

**Table 1:** The possible pathogenic mechanisms of different cytokines in *Staphylococcus aureus* enterotoxin B-induced CRS with nasal polyposis

Year, country and reference	Authors	Cell culture (target cytokine)	Outcome
2011, Canada [72]	Liu et al	Nasal mucosa (IL-17)	SEB may contribute to converting FoxP3+ Treg to FoxP3+ IL-17+ T cells. These cells may have a role in nasal polyposis by remodeling the nasal airways
2013, Japan [75]	Okano et al	Dispersed NP cells (IL-18)	Inhibition of IL-18 dramatically decreased the production of SEB-induced IFN-γ, IL-5, and IL-13. Thus, IL-18 may have pathogenic effects in CRS and nasal polyposis by boosting both the Th1 and Th2 responses
2014, Korea [76]	Jin et al	Human nasal epithelial cells (IL-17C)	Inflammatory conditions and bacterial challenges cause the production of IL-17C in epithelial cells. SEB induced the expression of this cytokine in nasal epithelial cells
2016, Japan [77]	Noyama et al	Dispersed NP and UT cells (IL-22)	SEB triggers IL-22 production from NP, and this cytokine may play a role in the pathogenesis of CRSwNP via its enhancement of MUC1
2016, Belgium [13]	Delemarre et al	PBMCs from healthy control and patients with CRSwNP (IL-9)	Stimulation with SEB and *S. aureus* leads to a remarkable increase in IL-9 gene expression
2017, Japan [78]	Haruna et al	Dispersed UT and NP cells from patients with CRSwNP and CRSsNP (IL-10)	SEB leads to the production of impaired IL-10 in NP, and this phenomenon may worsen the pathophysiology of CRS, such as lower airway obstruction and eosinophilia
2017, Sweden [79]	Calus et al	Nasal tissue of (IL-21)	SEB leads to the increase in IL-21 and IL-21+ CD4+ T cells, so; this enterotoxin may regulate the function of T-follicular helper cells in nasal polyps

CRSwNP, CRS with nasal polyps; CRSsNP, CRS without nasal polyps; NP, nasal polyp; UT, uncinate tissue; SEB, *Staphylococcus aureus* enterotoxin B; PBMCs, peripheral blood derived mononuclear

### Regulatory T cells (Tregs)

**Fig. 1:**
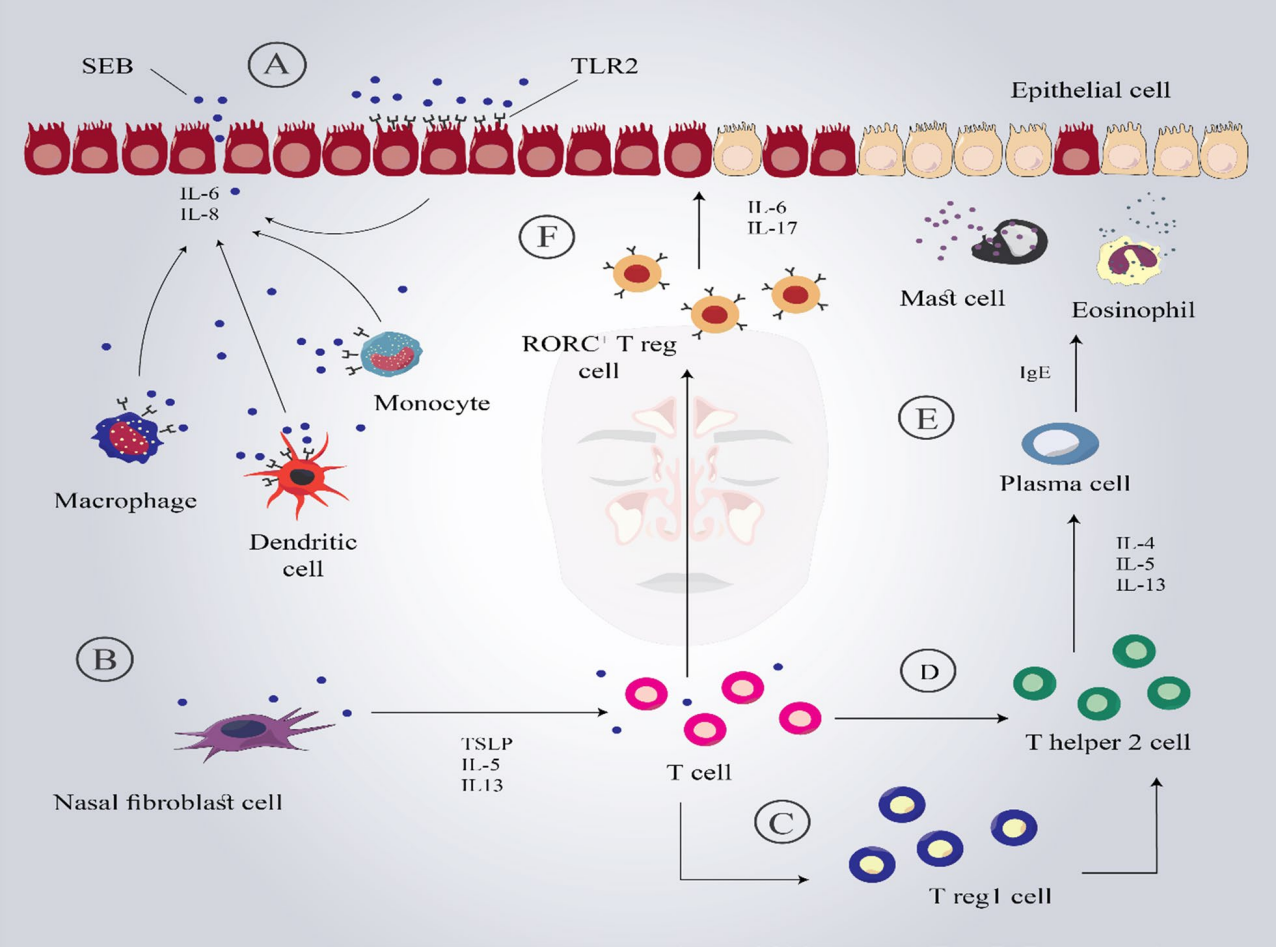
Possible mechanisms of SEB in inducing CRS and nasal polyposis. **A** SEB activates Toll Like Receptor 2 (TLR2) and triggers the production of pro-inflammatory cytokines; besides, it induces reactive oxygen species and endoplasmic reticulum stress-induced inflammation that may cause epithelial cell integrity disruption and enhance permeability. **B** Nasal fibroblast, especially nasal polyp fibroblast, and epithelial cells produce high levels of TSLP and result in Th2 differentiation and proliferation after exposure to SEB. **C** T regs 1 cell may reduce Th1 cells activity and increase the development of Th2 cells. **D** SEB-induced Typical type 2/Th2 pathway. **E** Th2-associated cytokines lead to the IgE production from plasma cells, eosinophils degranulation, the release of cationic proteins, and localized eosinophilic inflammation. **F** SEB could be involved in the further production of RORC+ Tregs, resulting in an increased production of inflammatory cytokines, which may contribute to the pathogenesis of eosinophilic nasal polyposis

In addition to Th2, a recently published examination reported the functional roles for different subsets of Tregs (Fig. 1). In this respect, a study conducted by Derycke et al. reported higher T cells in patients with CRSwNP compared to CRSsNP and controls. To this end, the T cell profile of control and CRSsNP samples was identical, whereas Th2 cells were significantly increased in CRSwNP mucosal inflammation. Notably, exposure to SEB induced T regulatory type 1 (Tr1) cells associated with Th2 bias in CRSwNP [68]. This is due to Tr1 cells having the potential to alter the Th1/Th2 balance by decreasing the activity of Th1 cells and increasing the development of Th2 cells [69, 70].

Another investigation also reported a subset of Tregs (CD4^+^CD25^high^FoxP3^+^T cells) that express IL-17 or Retinoic Acid Receptor Related Orphan Receptor C (RORC) (RORC^+^ Tregs or Th17-like Tregs). Due to the expression of IL-17 with the loss of suppressive function, these cells are considered new pro-inflammatory cells [71, 72]. In this regard, a previous study evaluated the SEB's role in differentiating Tregs to RORC^+^ Tregs. The findings demonstrated that RORC^+^ Tregs were significantly higher in the nasal polyps, especially in eosinophilic polyps, compared to the control mucosa. Intriguingly, after 24 h of stimulation of PBMC with SEB, significantly higher RORC^+^ Tregs were detected in the eosinophilic polyps group compared to the control group; however, this significant difference was not observed between noneosinophilic polyps and control groups. As a result, the authors proposed that RORC^+^ Tregs cells may be associated with eosinophilic nasal polyposis pathogenesis. Furthermore, SEB leads to the RORC+ Tregs differentiation and proliferation and higher production of IL-6 and IL-17, suggesting SEB may weaken the suppressive function against pathogenic T cells and be associated with tissue inflammation [71]. This supports the finding by Jin et al. that observed the higher expression of Hypoxia-Inducible Factor 1α (HIF-1α) and RORC in nasal polyps Tregs compared to the control mucosa [73]. It should be noted that HIF-1α enforce imbalance between Tregs and Th17 activity and could lead to autoimmune disorders and chronic inflammation [74]. In this regard, 24-h stimulation of PBMCs with SEB result in higher expression of HIF-1α and RORC in Tregs [73]. Thus, SEB may contribute to the continued production of RORC^+^ Tregs; consequently, a higher level of inflammatory cytokines in the presence of pathogenic T cells may contribute to the pathogenesis of nasal polyposis.

As demonstrated in the studies above, the SEB-induced typical type 2/Th2 pathway could predispose patients to CRSwNP. Furthermore, SEB may be involved in the higher expression of RORC and HIF-1α in Tregs, and maintaining the inflammation in sinonasal mucosa could lead to nasal polyposis' pathogenesis. In this respect, the development of new therapeutic agents that would decrease the number of Th2 cells, or redirect these Th2 cells into a Th1 phenotype, could be helpful for patients. However, in vivo studies using the animal model are required in the future to confirm the SEB-related pathogenesis of CRS and nasal polyps and to develop a possible therapeutic agent for nasal polyposis.

## Animal studies

In vivo analysis of SEB-induced nasal polyps and CRS remain unclear due to a lack of appropriate animal models. However, several in vitro findings have been confirmed in animal models, and this section discusses these studies.

New bone formation was detected in 36% of patients with primary CRS. It was reported that osteitis correlates with recalcitrant CRS, endoscopic sinus surgery, poor prognosis, nasal polyps, and eosinophilia in tissue [80–82]. Khalmuratova et al. instilled BALB/c male mice with 3% ovalbumin (OVA) plus 10 ng of SEB. Histopathologic analyses revealed thickened mucosae with polyp-like lesions, while the indication of inflammation was not detected in control mice treated with Phosphate-Buffered Saline (PBS). Furthermore, OVA/SEB-treated mice showed a higher number of osteoclasts and osteoblasts (at the rim of the bone) and irregular thickening of the sinus walls [80]. As a result, the authors proposed that the OVA/SEB challenge in mice leads to immune cells infiltration, periosteal thickening, and higher osteoblastic and woven bone formation activity. This study demonstrated a new sight of SEB pathogenesis in nasal polyps and CRS; however, confirming these findings requires further studies.

Rabbits were exposed to different bacterial toxins such as SEB in another examination. The results demonstrated that SEB contributes to CRS pathogenesis by inducing mucosal thickening and inflammatory cell infiltration (primarily eosinophils). Notably, this study did not use different doses of toxins to test dose-dependent responses; thus, the correlation between SEB doses and pro-inflammatory responses was not established. In summary, the authors proposed that persistent or prolonged exposure to bacterial toxins may be more relevant to CRS pathogenesis compared to acute and intense exposures [83].

In vitro studies reported that SEB leads to the typical type 2/Th2‐mediated inflammatory profile. The animal model also confirmed these findings. For instance, SEB and house dust mite (HDM) induced an allergic CRS mouse model. The results established that 22 weeks of HDM + SEB administration induces type 2/Th2 mediated inflammatory responses in mice. Additionally, this study demonstrated a decrease in the number and renewal of immature olfactory neurons without impairing mice's sense of smell as measured by electro-olfactograms and behavioral food tests [84]. Therefore, HDM + SEB-induced type 2/Th2 response could be associated with immature olfactory neurons decline. In this regard, not only physical characteristics like mechanical nasal obstruction could lead to olfactory loss, but also SEB-induced inflammatory responses might cause this phenomenon [84]. Another investigation also used the OVA/SEB to induce CRS in a murine model. Long-term instillation of OVA/SEB (24 weeks) results in nasal polyps, eosinophil infiltration, edematous mucosal thickness, and increased IL-10 levels compared to short-term treatment (12 weeks). Notably, eosinophils and neutrophils infiltration was observed in both duration of exposure, but eosinophil infiltration was higher in the long-term CRS model than in the short-term model. Furthermore, the expressions of TSLP and IL-25 were upregulated at mRNA and protein levels in long-term and short-term CRS models, respectively [85]. TSLP plays an essential role in promoting the Th2 and immune response cytokines and inhibiting IFN-γ production [86–88]. Nevertheless, the expression of IL-25 is associated with numerous inflammatory markers, including Th1/Th2/Th17 inflammation [88]. Additionally, Ahn et al. reported that four weeks of intranasal SEB administration to four-week-old Sprague–Dawley rats results in neutrophil infiltration in the lamina propria and the formation of neutrophil clusters in the sinus spaces. However, no evidence of eosinophilic inflammation was observed [89]. Thus, the studies above demonstrated that the duration of allergen exposure alters CRS features in a murine model, and prolonged exposure to the SEB result in Th2 skewed and dominant eosinophilic inflammation with an increased TSLP expression.

The pathogenic mechanisms of SEB in nasal polyposis were also investigated in different animal models. In one of these studies, OVA/SEB was instilled into the nasal cavity of mice to induce nasal polyps. The results indicated that the nasal lavage fluid contained increased levels of plasma and B cell markers, as well as IgG1 and IgA when compared to the control group [90]. Furthermore, in their allergic CRS murine model study, Khalmuratova et al. used HDM and SEB to induce allergic rhinosinusitis and nasal polyps. Nasal polypoid lesion formation and a notable increase in the number of eosinophilic infiltrate and secretory cells were detected in HDM + SEB-treated mice compared to the HDM-treated mice [91]. In another study, the authors used OVA and OVA plus SBE to induce nasal polyposis in a murine model. Compared to mice treated with OVA alone, more mucosal lesions with disrupted epithelial and nasal polypoid lesions were detected in OVA + SEB mice [18]. SEB-Th2-induced responses appear to result in the secretion of cytokines and the recruitment of eosinophils. Thus, intensive inflammation will disrupt epithelial cells and result in the formation of nasal polyps [65, 92]. Therefore, in combination with other allergens, SEB induces intense immune responses and may lead to nasal polyp formation. Generally, as reported through in vitro studies, animal models have also shown that immune responses stimulated by SEB can play a pivotal role in causing CRS and nasal polyposis, and knowing more about this pathway can be one of the critical strategies in managing this disease. Overall, the detailed analyses of SEB pathogenic mechanisms in animal models potentially open new perspectives to explain CRSwNP in humans.

## Inhibition of SEB-induced CRS and nasal polyposis

Understanding the interactions of SEB with different parts of immune systems and precise mechanisms of SEB-induced CRS and nasal polyposis can facilitate developing preventive and treatment approaches against CRSwNP. In this section, we will discuss different treatment approaches that have been used to inhibit SEB pathogenic mechanisms in CRS and nasal polyposis.

Bee venom (BV) is a traditional oriental medicine that has been used historically to treat malignant and chronic inflammatory diseases [93]. Shin et al. evaluated BV's anti-inflammatory effect on an allergic CRS mouse model. The authors induced allergic CRS in mice by injecting OVA with SEB into the nose. Afterward, they administered BV intranasally three times a week for eight weeks to treat the mice. Mice treated with BV showed lower periodic acid Schiff-positive cells and inflammatory cell infiltration. Eosinophil and neutrophil counts and INF-γ levels were significantly decreased in treated mice's nasal lavage fluid.

Moreover, BV significantly suppressed expressions of activator protein (AP)-1 and SEB-induced NF-κB in mouse nasal mucosa. Notably, treatment with a low concentration (0.5 or 5 ng/ml) of BV did not affect the nasal mucosa morphology and mouse survival, while higher concentrations of BV induced the production of chemical mediators from keratinocytes and nasal epithelial cells. The results of this study showed that BV reduced the production of INF-γ, mucin-producing cells, inflammatory cell infiltration, and Th1 cytokine in the nasal mucosa. The anti-inflammatory effects mentioned previously could result from the inhibition of AP1 and NF-κB and their associated pathways. Therefore, BV could be considered a therapeutic agent to improve the CRS inflammatory condition and can be used as an adjuvant agent that decreases adverse effects and increases the therapeutic potency of anti-inflammatory treatments [94]. However, further research is required to evaluate the best BV concentration for clinical usage and the anti-inflammatory features of each component of BV.

Yu et al. evaluated the anti-inflammatory effect of dexamethasone on SEB-induced pro-inflammatory mediators from human nasal epithelial cells. To this end, inferior turbinate and nasal polyp's epithelial cells were collected from subjects with obstructive sleep apnea syndrome without a history of CRSwNP and patients with CRSwNP, respectively. These cells were cultured serum-free under the stimulus of SEB; then, the anti-inflammatory effect of dexamethasone was assessed. The findings demonstrated that SEB increased the expression of the GM-CSF and IL-5 from human nasal epithelial cells. Notably, the expression of the mediators mentioned above was significantly higher in the epithelial cells of nasal polyps than in the inferior turbinate. Treatment with dexamethasone considerably suppressed the expression of SEB-induced GM-CSF and IL-5 [95].

In another examination, regulatory effects of dexamethasone and verapamil were studied in SEB-induced cytokines secretions. Verapamil, an L-type calcium channel blocker, has been shown to possess immunomodulatory characteristics in different tissues [96]. In this study, sinonasal polyp explants were isolated from 8 patients with CRSwNP; then, polyps were incubated for 24 h with verapamil or dexamethasone followed by an additional 24 h with SEB. Both verapamil and dexamethasone treatments significantly reduced SEB-stimulated IL-5 secretion. Verapamil significantly decreased SEB-stimulated IL-6 secretion, but not dexamethasone. Nevertheless, both treatments had no notable effects on SEB-stimulated TSLP secretion. In this respect, the findings of this study suggested that verapamil, with an efficacy approximating that of dexamethasone, can regulate the secretion of Th2-associated cytokine in organotypic sinonasal polyp explants. Hence, in addition to dexamethasone, verapamil also can be considered to inhibit inflammation caused by SEB in patients with CRSwNP [97].

Prostaglandin E2 (PGE2) is another potent inflammatory mediator used to inhibit CRSwNP resulting from SEB. In this regard, in the previous study, DNPC were challenged with SEB; then, the inhibitory effect of PGE2 on SEB-stimulated responses was evaluated, especially in terms of receptor specificity [98]. SEB led to the significant expression of RANTES, IL-5, and IL-13 from DNPC. Furthermore, PGE2 dramatically and dose-dependently decreased the production of mentioned mediators from DNPC in response to the SEB. Moreover, E-prostanoid (EP) 2 receptor-selective agonist predominantly inhibited the production of all of the cytokines mentioned above. Thus, PGE2 regulates the eosinophilia-associated cytokine/chemokine production by DNPCs in response to SEB via the EP2-mediated pathway. These data suggest that local or systemic administration of PGE derivatives like misoprostol could be impressive for managing eosinophilic airway diseases, such as bronchial asthma, allergic rhinitis, and CRSwNP [98].

Finally, Paramasivan et al. evaluated the anti-inflammatory effect of chitosan on SEB-induced cytokine from human nasal fibroblast [99]. Chitosan's ability to regulate the expression of Th2 pro-inflammatory cytokines and its newly discovered antimicrobial property has prompted additional research into its use in managing a chronic inflammatory disease caused by bacterial infection [100, 101]. To this end, the authors isolated fibroblasts cells from human nasal tissue and then challenged these cells with SEB. Chitosan significantly reduced the expression of IL-8 after the SEB challenge. Notably, chitosan effectively reduced the expression of IL-8 expression in non-challenged fibroblasts showing its anti-inflammatory effects on fibroblasts [99].

Chronic inflammation reduction of the sinonasal mucosa is one of the main challenges in treating patients with CRS. Inflammation management provides an opportunity for the sinonasal cavity lining to re-epithelialize and re-establish functional cilia activity [102]. SEB-induced pro-inflammatory cytokines in sinonasal mucosa are one of the key factors for predisposing patients to CRSwNP. As mentioned previously, anti-inflammatory agents such as BV, chitosan–dextran gel, PGE2, dexamethasone, and calcium channel blockers have been shown to inhibit SEB-induced pro-inflammatory cytokines. Although some studies reported promising results, additional animal models and clinical trials are needed before pursuing the use of these agents as a localized therapy for CRS.

## Conclusion

In previous research, SEB was implicated as a risk factor for CRS and nasal polyposis. Chronic inflammation and sinonasal mucosal damage caused by SEB have been identified as the primary mechanisms underlying CRS; however, the precise SEB pathogenic mechanisms remain unknown. Furthermore, many of the functional roles of SEB in CRS have not been established in animal models. As a result, additional research is required to ascertain the precise mechanism of SEB pathogenesis in CRS and nasal polyposis, and once these mechanisms are identified, therapeutic strategies can be developed to prevent and even treat CRS.

## Data Availability

The authors confirm that the data supporting the findings of this study is available within the article.

## Abbreviations

CRS: Chronic rhinosinusitis
CRSsNP: CRS without nasal polyps
CRSwNP: CRS with nasal polyps
SEB: *Staphylococcus aureus* Enterotoxin B
ROS: Reactive oxygen species
